# Increased Diagnostic Accuracy of Digital vs. Conventional Clock Drawing Test for Discrimination of Patients in the Early Course of Alzheimer’s Disease from Cognitively Healthy Individuals

**DOI:** 10.3389/fnagi.2017.00101

**Published:** 2017-04-11

**Authors:** Stephan Müller, Oliver Preische, Petra Heymann, Ulrich Elbing, Christoph Laske

**Affiliations:** ^1^Department of Psychiatry and Psychotherapy, Eberhard Karls UniversityTübingen, Germany; ^2^Geriatric Center at the University Hospital, Eberhard Karls UniversityTübingen, Germany; ^3^German Center for Neurodegenerative Diseases (DZNE)Tübingen, Germany; ^4^Section for Dementia Research, Hertie Institute for Clinical Brain Research and Department of Psychiatry and Psychotherapy, Eberhard Karls UniversityTübingen, Germany; ^5^Art Therapy Research Institute, Nürtingen-Geislingen UniversityNürtingen, Germany

**Keywords:** early Alzheimer’s disease, amnestic mild cognitive impairment, screening, clock drawing test, online-drawing

## Abstract

The conventional Clock Drawing Test (cCDT) is a rapid and inexpensive screening tool for detection of moderate and severe dementia. However, its usage is limited due to poor diagnostic accuracy especially in patients with mild cognitive impairment (MCI). The diagnostic value of a newly developed digital Clock Drawing Test (dCDT) was evaluated and compared with the cCDT in 20 patients with early dementia due to AD (eDAT), 30 patients with amnestic MCI (aMCI) and 20 cognitively healthy controls (HCs). Parameters assessed by dCDT were time while transitioning the stylus from one stroke to the next above the surface (i.e., time-in-air), time the stylus produced a visible stroke (i.e., time-on-surface) and total-time during clock drawing. Receiver-operating characteristic (ROC) curves were calculated and logistic regression analyses have been conducted for statistical analysis. Using dCDT, time-in-air was significantly increased in eDAT (70965.8 ms) compared to aMCI (54073.7 ms; *p* = 0.027) and HC (32315.6 ms; *p* < 0.001). In addition, time-in-air was significantly longer in patients with aMCI compared to HC (*p* = 0.003), even in the aMCI group with normal cCDT score (54141.8 ms; *p* < 0.001). Time-in-air using dCDT allowed discrimination of patients with aMCI from HCs with a sensitivity of 81.3% and a specificity of 72.2% while cCDT scoring revealed a sensitivity of 62.5% and a specificity of 83.3%. Most interestingly, time-in-air allowed even discrimination of aMCI patients with normal cCDT scores (80% from all aMCI patients) from HCs with a clinically relevant sensitivity of 80.8% and a specificity of 77.8%. A combination of dCDT variables and cCDT scores did not improve the discrimination of patients with aMCI from HC. In conclusion, assessment of time-in-air using dCDT yielded a higher diagnostic accuracy for discrimination of aMCI patients from HCs than the use of cCDT even in those aMCI patients with normal cCDT scores. Modern digitizing devices offer the opportunity to measure subtle changes of visuo-constructive demands and executive functions that may be used as a fast and easy to perform screening instrument for the early detection of cognitive impairment in primary care.

## Introduction

The number of cognitively impaired individuals will increase dramatically as the elderly population increases. Thus, the issue of screening for dementia and cognitive impairment will become increasingly important. However, currently nearly 50% of dementia cases are not diagnosed (Boustani et al., 2003; Mukadam et al., 2015). In addition, there is a considerable delay in the diagnosis of dementia which may reduce the effectivity of available treatments (Boustani et al., 2003; Carpentier et al., 2010; Rapp, 2014; Akl et al., 2015). Furthermore, currently available measures for diagnosis of dementia are time-consuming (psychometric testing), invasive (cerebrospinal fluid (CSF) examination) or expensive (neuro-imaging). Thus, there is an urgent need to develop fast and easily to perform, non-invasive and non-expensive diagnostic measures able to accurately detect people with cognitive impairment and dementia in the earlier stages to provide them further diagnostics (i.e., CSF examination and/or neuro-imaging) in case of positive screening results. This may be beneficial to allow earlier onset of available treatment medications and to allow more careful planning of financial and support systems when the patients are still in a position to make their wishes known (Solomon and Murphy, 2005).

The basic purpose of cognitive screening tests is to indicate the likelihood of genuine cognitive impairment, inferred from the relationship of the patient’s score to reference norms. A very impaired score (along with supporting history) may lead a physician to make a diagnosis without further investigation; a borderline score may prompt referral for specialist assessment (e.g., at a memory clinic), where available. The success of a particular screening tool for this purpose lies in its statistical robustness—ideally, high sensitivity and specificity along with a high positive predictive value in a population with a relevant base rate of impairment.

In this context the conventional Clock Drawing Test (cCDT) is one of the most widely used tests for screening cognitive impairment and dementia that has been well accepted among clinicians and patients for its ease of use and short administration time (Shulman, 2000). The cCDT is a valuable cognitive screening test for both quantitative and qualitative assessment of a variety of cognitive functions, including selective and sustained attention, auditory comprehension, verbal working memory, numerical knowledge, visual memory and reconstruction, visuospatial abilities, on-demand motor execution (praxis) and executive function (Shulman et al., 1986). The ease of use and broad range of cognitive abilities required for successful completion of the cCDT have made this an effective and increasingly popular cognitive screening instrument among researchers and clinicians, however interpretation of the cCDT necessitates consideration of the wide range of cognitive abilities that are assessed by this test (Frey and Arciniegas, 2011). Furthermore, although there is great interest in the cCDT as a cognitive screening tool, there are multiple cCDT administration and scoring systems with no consensus on which system produces the most valid results while remaining user friendly (Ricci et al., 2016). Additionally, while there is a general agreement in considering the cCDT as a useful test to detect moderate and severe dementia (Pinto and Peters, 2009), its utility in distinguishing patients with mild cognitive impairment (MCI) or mild dementia is still debated (Seigerschmidt et al., 2002; Pinto and Peters, 2009; Ehreke et al., 2011).

cCDT demands like drawing and handwriting are complex human activities that entail an intricate blend of cognitive, kinesthetic and perceptual-motor components, including visual perception, memory and reconstruction, visuospatial abilities, on-demand motor planning and execution (praxis) and executive function (Forbes et al., 2004; Yan et al., 2008; Forbes-Mckay et al., 2014). Thus, these characteristics of the drawing process suggest that it might be sensitive to impairments in cognitive functioning, and thus assessments of drawings might facilitate the diagnosis of such impairments (Schmitt et al., 2009). Indeed, deficits in these areas reflect possible frontal and temporoparietal disturbances that are often exhibited in AD (Samton et al., 2005), and that may not easily be detected by commonly-used cognitive screening tests such as the Mini-Mental State Examination (MMSE; Brodaty and Moore, 1997). Furthermore, disturbances in the sensory-motor system due to neuropathological changes may contribute to reduced levels of motor performance associated with Alzheimer’s disease (AD; Dick et al., 1995; Walker et al., 1997; Petersen et al., 2000; Pennanen et al., 2004). However, although there may be some generalizations about what can be considered a normal clock, it is important to note that if a subject’s clock shows an error, it does not necessarily mean that the person is impaired. Likewise, if a clock drawing falls into the range of “normal” performance, this does not necessarily implicate that the subject is cognitively normal.

Modern digitizing tablets not only gather the *x-y* coordinates that describe the movement of the writing device as it changes its position during handwriting or drawing, but they can also take into account information collected when the writing device is not exerting pressure on the writing surface (i.e., in-air movements performed by the hand while transitioning from one stroke to the next; during these movements the writing device exerts no pressure on the surface). Recent experimental results based on pen pressure and in-air movements appear to be significantly different for patient with early AD compared to healthy control (HC) individuals (Faundez-Zanuy et al., 2014). More recently, the diagnostic value of a tablet-based drawing task (i.e., drawing three-dimensional house) for the discrimination of patients with amnestic MCI (aMCI) or early dementia of Alzheimer-type (eDAT) from HC could be demonstrated (Müller et al., 2017). In this study i.e., time-in-air differed significantly between patients with aMCI, eDAT and HCs demonstrating an excellent sensitivity and a moderate specificity to discriminate aMCI subjects from normal elderly and an excellent sensitivity and specificity to discriminate patients affected by mild AD from healthy individuals (Müller et al., 2017).

Other studies reported increased writing thickness and pressure of the pen in patients with mild to moderate dementia due to AD compared with HCs (Labarge et al., 1992). Moreover, patients who were either apractic from an early stage of AD or who switched to being apractic as the disease progressed had a faster rate of decline than those patients who remained able to complete drawing tasks (Smith et al., 2001). Thus, patients who were unable to complete the praxis task from an early stage had a worse prognosis than those who maintained this ability. In line with this it was found that handwriting difficulties were correlated with the severity of AD and the concomitant cognitive impairment (Schröter et al., 2003). It seems that motor skills were impaired early in AD because they require cognitive decision making in order to perform automatic retrieval (Croisile, 1999). Kinematical results of circular and quick handwriting movements performed by patients with AD, MCI and HCs showed a greater variability in movement velocity in AD and MCI patients (Schröter et al., 2003).

In the present study we evaluated the diagnostic value of a newly developed digital Clock Drawing Test (dCDT) assessing time-in-air, time-on-surface and total-time of the drawing process in patients with aMCI, eDAT and HCs and compared it with the diagnostic value of cCDT (Shulman, 2000).

## Materials and Methods

### Participants

Our study included 70 participants (34 females and 36 males) with a mean age of 66.9 ± 10.3 years. All participants were right-hander (Oldfield, 1971), had normal or corrected-to-normal visual acuity and sufficient hearing ability. No participants had a physical handicap that affected his or her ability to perform the tasks or any indication of other neurological or psychiatric disorders unrelated to his or her diagnosis. To exclude symptoms of depression, all participants completed the German 15-item version of the Geriatric Depression Scale (GDS; exclusion criterion GDS > 5; Yesavage et al., 1982). The local ethical committee at the University Hospital of Tübingen approved the study. All participants signed an informed consent form after receiving a detailed explanation of the study.

### Patients with aMCI or eDAT

Patients with aMCI or eDAT were recruited from the Memory Clinic of the Department of Psychiatry and Psychotherapy at the University Hospital of Tübingen. They underwent physical, neurological, neuropsychological and psychiatric examinations as well as brain imaging. Routine laboratory tests included Lues serology and analysis of vitamin B12, folic acid and thyroid-stimulating hormone levels.

The diagnostic criteria for eDAT were defined according to the National Institute of Neurological and Communicative Disorders and Stroke Alzheimer’s Disease and Related Disorders Association (McKhann et al., 1984; Petersen et al., 2010). All of these patients had a score of 4 on the Global Deterioration Scale (Reisberg et al., 1982).

The diagnosis of aMCI was defined according to the Mayo criteria (Petersen, 2004), which include the presence of a memory complaint (corroborated by an informant), objectively impaired memory function, intact activities of daily living and the absence of dementia.

### Healthy Control Group

HC individuals had no history of neurological or psychiatric disease or any sign of cognitive decline, as confirmed by a clinical interview.

### Neuropsychological Assessment

All participants undergo a neuropsychological examination that includes the German version of the modified Consortium to Establish a Registry for Alzheimer’s Disease neuropsychological test battery (CERAD; Morris et al., 1989) including the MMSE (Folstein et al., 1975) and the Trail Making Test (Reitan, 1958).

The Trail Making Test part A (TMT-A) assesses psychomotor speed operationalized by connecting as fast as possible the numbers 1–25 in ascending order. Trail Making Test part B (TMT-B) includes numbers (1–13) and letters (A–L) which must be connected in an ascending alternating pattern (i.e., 1-A-2-B-3-C, etc.) as fast as possible. As the subjects have to switch between mental sets the TMT-B is used to assess cognitive flexibility and reflects executive functions (Crowe, 1998; Chen et al., 2009).

### Computerized Assessment of Clock Drawing

A Windows Surface Pro 4 digitizer and a handheld stylus pen were used to assess drawing movements. The tablet acquires 120 samples per second including the spatial coordinates (*x, y*). The spatial accuracy is 0.25 mm (resolution 267 pixels per inch). Binary variables indicating pen-up state (i.e., 0) or pen-down state (i.e., 1).

Time-in-air and time-on-surface are assessed in milliseconds (ms) according to their binary coding, total-time correspond to the time-in-air plus time-on-surface. All participants performed the cCDT on the digitizer with a handheld stylus pen following the instruction to draw a circle (i.e., clock face) with the numbers in appropriate positions and to place the hands on the clock to indicate “10 past 11 o’clock”. The results were evaluated by scoring from 1 point (i.e., perfect) to 6 points (i.e., not representative of a clock at all). A score ≥3 was considered as impaired (Shulman, 2000).

### Data Analysis

The statistical software package SPSS (version 23) was used for data analyses. For all tests, the level of statistical significance was set to *p* < 0.05. Levene’s test served to assess homogeneity of variances. We used the Pearson chi-square test to detect group differences in gender distribution and the nonparametric Kruskal-Wallis test to detect group differences in cCDT and GDS scores. Group differences in age, education and global cognition (MMSE), TMT-A and B, time-in-air, time-on-surface and total-time were assessed using one-way analysis of variance (ANOVA) followed by a *post hoc* Tukey test. To asses group differences in clinical and demographical variables (i.e., age, years of education and MMSE), TMT-A and B, as well as in time-in-air, time-on-surface and total-time between patients with aMCI showing normal cCDT-scores (i.e., cCDT score of 1 or 2) and healthy individuals *t*-tests for independent samples were conducted. Bonferroni adjustment was used to correct for type I errors introduced by the use of multiple tests (i.e., comparisons for this analysis were performed at the *p* < 0.0062 level of significance).

Receiver operating characteristics (ROC) curves were established to illustrate the specificity of dCDT variables (i.e., time-in-air, time-on-surface and total-time) as well as cCDT scores in relation to the sensitivity in classifying HC individuals and patients with aMCI (HCs vs. patients with eDAT, respectively).

To evaluate the utility of the target variables in distinguishing HCs and patients with aMCI (HCs vs. patients with eDAT, respectively) logistic regression analyses were conducted, with diagnostic group as the dependent variable and time-in-air, time-on-surface, total-time and cCDT scores as the independent variable, adjusted for age, gender and years of education and controlled for the influence of psychomotor speed and executive functions (i.e., TMT scores). To evaluate diagnostic accuracy of dCDT variables in case of a subjects’ cCDT-score indicates no impairment after visual inspection (i.e., CDT score 1 or 2) the same analyses (i.e., ROC curves and logistic regression analyses) have been performed for patients with aMCI showing normal cCDT scores and healthy individuals.

In order to investigate whether the combined analysis of time-in-air, time-on-surface, total-time and cCDT scores would improve the discrimination of patients with aMCI from controls in terms of sensitivity and specificity logistic regression models were constructed in which we combined the predictor variables. Time-in-air, time-on-surface, total-time and cCDT scores (without cCDT scores for patients with aMCI showing normal cCDT values and healthy individuals respectively) were entered into a forward stepwise logistic regression model based on significant improvement in Log Likelihood Ratios and significance of the included variables testing their ability to predict aMCI (with impaired cCDT scores likewise normal cCDT scores) and HC in the derivation cohort. Predictor variables were adjusted for age, gender and years of education and selected for inclusion in the model if *p* < 0.05.

## Results

### Clinical and Demographic Characteristics of the Participants

HC, aMCI and AD groups did not significantly differ in age, education and gender distribution. Also, GDS scores were not significantly different between groups and well below the cut-off score of 6. As expected, a one-way ANOVA revealed highly significant differences in MMSE scores (*F*_(2,67)_ = 104.85; *p* < 0.001). HC subjects had higher mean MMSE scores than aMCI (*p* < 0.001) and AD patients (*p* < 0.001). Moreover, mean MMSE scores of aMCI patients were higher than those of AD patients (*p* < 0.001). Performance in TMT-A (*F*_(2,67)_ = 12.26; *p* < 0.001) as well as TMT-B (*F*_(2,67)_ = 38.03; *p* < 0.001) differed significantly between the groups (Table 1). *Post hoc* Tukey analysis revealed that time to complete TMT-A and B was significantly longer (indicating greater impairment) in the eDAT group compared to HCs (TMT-A and B: *p* < 0.001) and aMCI patients (TMT-A: *p* = 0.003; TMT-B: *p* < 0.001). Healthy individuals and patients with aMCI did not differ significantly in either TMT-A (*p* = 0.121) or TMT-B (*p* = 0.186).

**Table 1:** **Clinical and demographic characteristics of HC individuals, patients with aMCI and patients with early dementia due to AD (eDAT)**.

	Group			*p*-value
	HC	aMCI	eDAT
*n*	20	30	20
Age in years	66.9 (9.4)	65.3 (6.6)	69.6 (6.1)	0.368
Years of education	13.2 (3.2)	11.9 (2.7)	11.8 (3.0)	0.230
Gender (M/F)	12/8	15/15	9/11	0.624
GDS	2.7 (2.2)	2.1 (2.0)	2.4 (1.7)	0.345
MMSE	29.4 (0.6)	26.6 (1.6)	21.7 (3.4)	<0.001
TMT-A	38.65 (10.8)	53.52 (24.5)	78.7 (36.7)	<0.001
TMT-B	85.0 (32.0)	108.55 (54.5)	203.55 (44.2)	<0.001
cCDT	1.2 (0.4)	1.9 (0.8)	2.9 (0.9)	<0.001
no. CDT Score 1 (%)	16 (80.0)	11 (36.7)	2 (10.0)
no. CDT Score 2 (%)	4 (20.0)	13 (43.3)	2 (10.0)
no. CDT Score 3 (%)	0 (0.0)	6 (20.0)	11 (55.0)
no. CDT Score 4 (%)	0 (0.0)	0 (0)	5 (25.0)
Time-in-air (ms)	32315.6 (5633.8)	54073.7 (18174.9)	70965.8 (34843.6)	<0.001
Time-on-surface (ms)	15712.8 (3434.5)	19332.5 (6416.9)	18233.9 (4337.8)	= 0.056
Total-time (ms)	49228.4 (10358.1)	73309.4 (20952.9)	86199.8 (40226.7)	<0.001

*Note: Values are expressed as mean (standard deviation). n, number; HC, healthy control individuals; aMCI, amnestic mild cognitive impairment; eDAT, early Alzheimer-type dementia; M/F, male/female; GDS, Geriatric Depression Scale (higher score indicates more severe depressive symptoms; maximum 15; scores of >5 indicates depression); MMSE, Mini Mental State Examination; TMT, Trail Making Test (part A or B, time in seconds); cCDT, conventional Clock Drawing Test (a score ≥3 is considered as impaired); %, percentage; ms, time in milliseconds*.

Comparing healthy individuals and the aMCI group with normal cCDT score (i.e., a cCDT score of 1 or 2) the HC group had significantly higher mean MMSE scores compared to patients with aMCI (*p* < 0.001), but they did not differ in TMT-A or B performance, age, education and gender distribution (Table 2).

**Table 2:** **Clinical and demographic characteristics of HC individuals and patients with aMCI with normal cCDT scores**.

	Group		*p*-value
	HC	aMCI
*n*	20	24
Age in years	66.9 (9.4)	65.9 (8.5)	=0.836
Years of education	13.2 (3.2)	12.5 (2.5)	=0.170
Gender (M/F)	12/8	12/12	=0.556
MMSE	29.4 (0.6)	27.1 (1.6)	<0.001
TMT-A	38.65 (10.8)	45.42 (23.8)	=0.089
TMT-B	85.0 (32.0)	95.08 (35.5)	=0.336
cCDT	1.2 (0.4)	1.5 (0.5)	=0.031
no. CDT Score 1 (%)	16 (80.0)	11 (45.8)	
no. CDT Score 2 (%)	4 (20.0)	13 (54.2)	
Time-in-air (ms)	32315.6 (5633.8)	54141.8 (17428.1)	<0.001
Time-on-surface (ms)	15712.8 (3434.5)	19990.1 (6836.3)	= 0.011
Total-time (ms)	49228.4 (10358.1)	74133.1 (20254.3)	<0.001

*Note: Values are expressed as mean (standard deviation). n, number; HC, healthy control individuals; aMCI, amnestic mild cognitive impairment; M/F, male/female; GDS, Geriatric Depression Scale (higher score indicates more severe depressive symptoms; maximum 15; scores of >5 indicates depression); MMSE, Mini Mental State Examination; TMT, Trail Making Test (part A or B, time in seconds); cCDT, conventional Clock Drawing Test (a score ≥3 is considered as impaired); %, percentage; ms, time in milliseconds*.

### Performance on the Clock Drawing Task in Patients with aMCI, eDAT and Healthy Individuals

Overall, cCDT performance differed significantly between the groups (*χ*^2^_(2)_ = 31.525; *p* < 0.001; Table 1). cCDT scores were significantly higher (i.e., indicating greater impairment) in patients with eDAT compared to MCI (*p* < 0.001) and HC (*p* < 0.001) as well as in patients with aMCI compared to healthy individuals (*p* = 0.009).

Analyzing the digitized variables during clock drawing, we found a significant difference in time-in-air (*F*_(2,67)_ = 15.230; *p* < 0.001; Table 1). Transitioning the drawing hand from one stroke to the next requires significantly less time in the HC group compared to patients with aMCI (*p* = 0.003) and eDAT (*p* < 0.001). Additionally, time-in-air in the aMCI group was significantly shorter compared to patients with eDAT (*p* = 0.027).

Although a stable trend was observable time-on-surface just failed to differ between the groups on the predetermined significance level (*F*_(2,67)_ = 3.008; *p* = 0.056; Table 1).

Finally, we found significant differences in total-time (*F*_(2,67)_ = 10.518; *p* < 0.001; Table 1). HC individuals required significantly less time to complete clock drawing compared to the aMCI (*p* = 0.005) and the eDAT group (*p* < 0.001). However, patients with aMCI and eDAT did not differ significantly (*p* = 0.202).

### Screening Value of the Clock Drawing Task in Patients with aMCI, eDAT and Healthy Individuals

**Figure 1:**
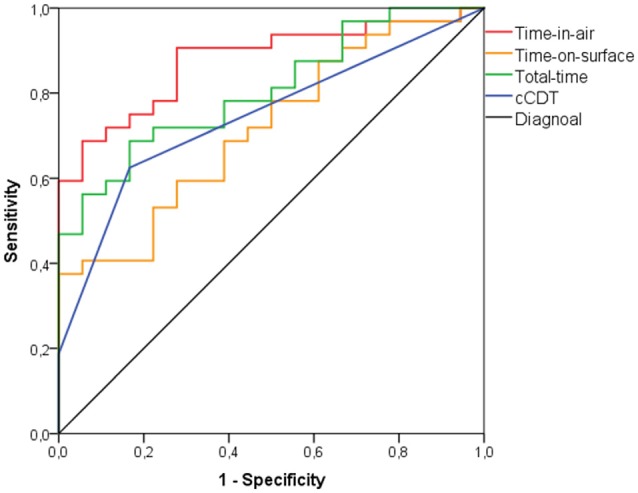
**Receiver-operating characteristic (ROC) curve analysis for discriminating patients with amnestic mild cognitive impairment (aMCI) from healthy control individuals (HC) using time-in-air, time-on-surface, total-time, or conventional clock drawing test (cCDT) scores**.

**Figure 2:**
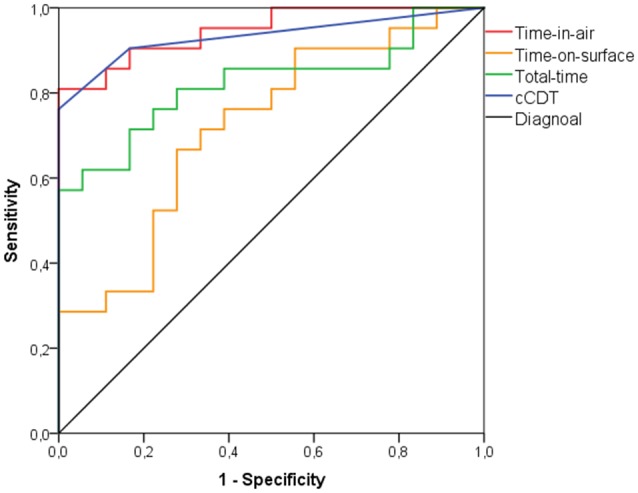
**ROC curve analysis for discriminating patients with early dementia due to Alzheimer’s disease (eDAT) from HC individuals using time-in-air, time-on-surface, total-time, or cCDT scores**.

When the traditional scoring system of the cCDT was used (i.e., a score ≥3 is considered as impaired) to discriminate between the aMCI group and healthy individuals, the best cut-off was a score of 1.5 (AUC: 0.745; *p* = 0.004; Figure 1) revealing an accuracy of 70.0% with sensitivity of 62.5% and specificity of 83.3% (Table 3). With an accuracy of 87.1% in discriminating the eDAT group and HCs a score of 2.5 (AUC: 0.933; *p* < 0.001; Figure 2) on the cCDT had a sensitivity of 90.5% and a specificity of 83.3% (Table 3).

**Table 3:** **Diagnostic value of time-in-air, time-on-surface, total-time and cCDT scoring in differentiating patients with aMCI or eDAT from HC individuals HC**.

	HC vs. aMCI			HC vs. eDAT		
	Sensitivity	Specificity	Accuracy	Sensitivity	Specificity	Accuracy
Time-in-air	81.3	72.2	78.0	85.7	88.9	87.2
Time-on-surface	75.0	50.0	66.0	76.2	61.1	69.2
Total-time	71.9	61.1	68.0	71.4	83.3	76.9
cCDT	62.5	83.3	70.0	90.5	83.3	87.1

*HC, healthy control individuals; aMCI, amnestic mild cognitive impairment; eDAT, early dementia due to Alzheimer’s disease; cCDT, conventional Clock Drawing Test*.

An accuracy of 78.0% was found when time-in-air was used to discriminate between the aMCI group and healthy individuals at 35588 ms (AUC: 0.880; *p* < 0.001; Figure 1) with sensitivity of 81.3% and specificity of 72.2% (Table 3). Between the eDAT group and HCs a cut-off of 39869 ms (AUC: 0.947; *p* < 0.001; Figure 2) on the time-in-air score revealed an accuracy of 87.2% with a sensitivity of 85.7% and a specificity of 88.9% (Table 3).

When total-time was used to discriminate between the aMCI group and healthy individuals, the best cut-point was at 51187 ms total-time (AUC: 0.813; *p* < 0.001; Figure 1) showing an accuracy of 68.0% with a sensitivity of 71.9% and specificity of 61.1% (Table 3). Between the eDAT group and HCs a cut-off of 58165 ms (AUC: 0.823; *p* = 0.001; Figure 2) on the total-time score had an accuracy of 76.9%, a sensitivity of 71.4% and a specificity of 83.3% (Table 3).

As time-on-surface failed to differ between the groups significantly this variable was excluded from further discrimination analysis.

In the first step (model 1), time-in-air was included in the forward stepwise logistic regression model and finally cCDT scores (model 2). Model 2 yielded significant Odds Ratios (95% confidence interval) for time-in-air (6.515 [6.463–6.567]; *p* = 0.004) and cCDT scores (4.671 [4.567–4.781]; *p* = 0.046).

Time-in-air (i.e., model 1) explained 27.9% of the variance (adjusted *R*^2^ = 0.279, *F*_(1,49)_ = 18.587, *p* < 0.001). Model 2 (i.e., time-in-air and cCDT scores) explained 35.6% of the variance (adjusted *R*^2^ = 0.356, *F*_(2,49)_ = 12.965, *p* < 0.001; *R*^2^ change = 0.076, *F*_(1,47)_ = 5.572; *p* = 0.022).

Time-on-surface (*p* = 0.271) and total-time (*p* = 0.583) were excluded from the regression model. However, with an accuracy of 76.0% resulting from 81.3% sensitivity and 66.7% specificity, the combination of time-in-air and cCDT scores did not improve the discrimination of patients with aMCI from HCs.

### Performance on the Clock Drawing Task in Patients with aMCI Showing Normal cCDT Scores and Healthy Controls

Between patients with aMCI (*n* = 24) with normal cCDT scores (i.e., cCDT score of 1 or 2) and healthy individuals (*n* = 20) time-in-air (*p* < 0.001), and total-time (*p* < 0.001) were significantly lower in HC individuals compared to patients with aMCI. Time-on-surface (*p* = 0.011) did not differ with respect to the adjusted significance level (i.e., *p* < 0.0062; Table 2).

### Screening Value of the Clock Drawing Task in Patients with aMCI Showing Normal cCDT Score and Healthy Controls

**Figure 3:**
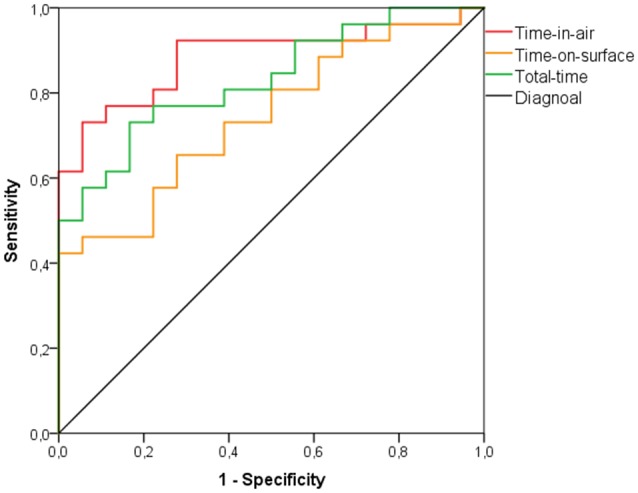
**ROC curve analysis for discriminating patients with aMCI with unimpaired cCDT scores (i.e., cCDT score 1 or 2) from HC individuals using time-in-air, time-on-surface, or total-time**.

In the aMCI group with normal cCDT scores and HC time-in-air discriminated best with an accuracy of 79.5% at a cut-off of 36468 ms (AUC: 0.885; *p* < 0.001; Figure 3) showing a sensitivity of 80.8% and a specificity of 77.8% (Table 4).

**Table 4:** **Diagnostic value of time-in-air, time-on-surface and total-time in differentiating patients with aMCI and HC individuals with unimpaired cCDT scores (i.e., a cCDT score of 1 or 2)**.

	HC vs. aMCI		
	Sensitivity	Specificity	Accuracy
Time-in-air	80.8	77.8	79.5
Time-on-surface	65.4	61.1	63.6
Total-time	76.9	72.2	75.0

*HC, healthy control individuals; aMCI, amnestic mild cognitive impairment*.

An accuracy of 63.6% was found when time-on-surface was used to discriminate between aMCI patients with normal cCDT score and HCs at a cut-point of 15803 ms (AUC: 0.744; *p* = 0.007; Figure 3) with sensitivity of 65.4% and specificity of 61.1% (Table 4).

When total-time was used to discriminate between the aMCI group with normal CDT scores and healthy individuals, an accuracy of 75.0% was found at 54624 ms (AUC: 0.831; *p* < 0.001; Figure 3) with sensitivity of 76.9% and specificity of 72.2% (Table 4).

In order to improve the discrimination of aMCI with normal cCDT from HC forward stepwise logistic regression model combing time-in-air, time-on-surface and total-time revealed significant Odds Ratio (95% confidence interval) for time-in-air as the best predictor variable (6.858 [6.807–6.909]; *p* = 0.005). The combination with time-on-surface (*p* = 0.221), and/or total-time (*p* = 0.853) did not improve discriminative power and were excluded from the model assumptions.

**Figure 4:**
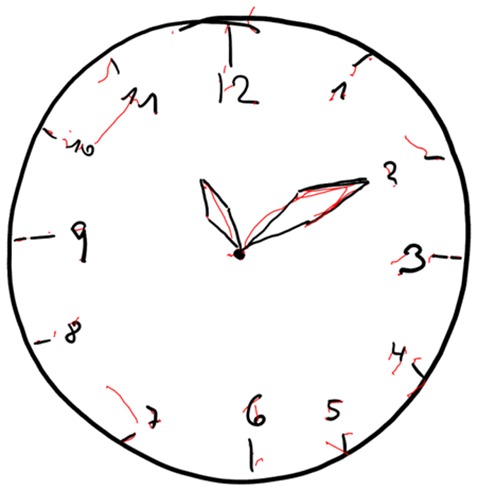
**Unimpaired clock drawing performed by a HC individual.** On-surface curves (i.e., pen-down) are displayed in deep black color, in-air movements are displayed in red color.

**Figure 5:**
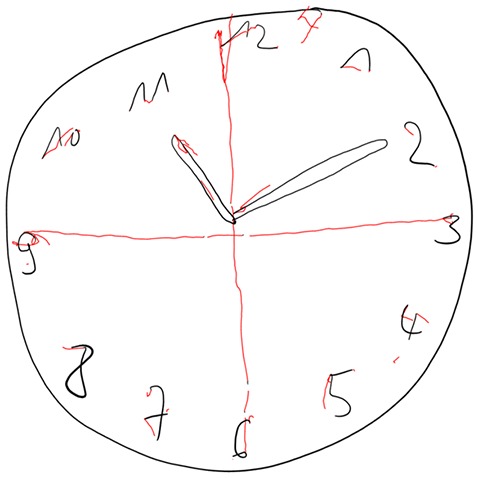
**Unimpaired clock drawing performed by a patient with aMCI.** On-surface curves (i.e., pen-down) are displayed in deep black color, in-air movements are displayed in red color.

**Figure 6:**
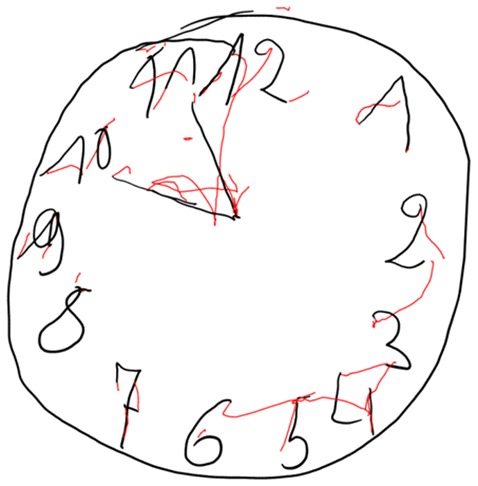
**Impaired clock drawing performed by a patient with aMCI.** On-surface curves (i.e., pen-down) are displayed in deep black color, in-air movements are displayed in red color.

**Figure 7:**
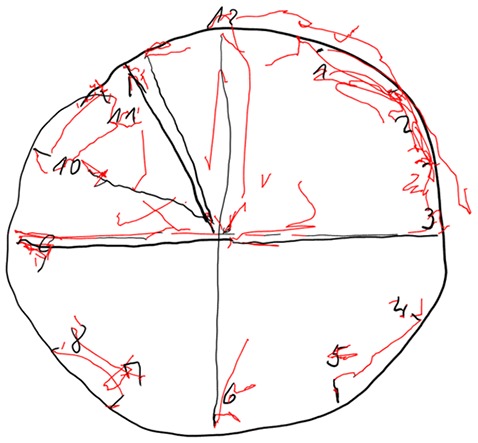
**Impaired clock drawing performed by a patient with eDAT.** On-surface curves (i.e., pen-down) are displayed in deep black color, in-air movements are displayed in red color.

In Figures 4–7 we present clock drawing (i.e., “ten past eleven”) performed by a healthy individual (Figure 4), two patients with aMCI (Figures 5, 6) and a patient with eDAT (Figure 7). The visual inspection of the on-surfaces curves (deep back color) of Figure 5 suggests an unimpaired clock drawing test score. However, the visual information provided by the in-air movements (red color) indicates that the patient drew in-air spokes to orient spacing. In Figures 6, 7 the minute hand points to 10 with progressive impairment and disorganization in the eDAT patient (i.e., Figure 7).

## Discussion

In the present study, we examined the possible influence of aMCI and early dementia development on alterations in movement execution (i.e., time-in-air, time-on-surface and total-time) during online CDT performance and whether these variables or their combination could be used to discriminate patients in the early course of AD (aMCI and mild dementia due to AD) from healthy individuals. We further compared these results with the sensitivity and specificity of the established Shulman’s CDT scoring method for its screening value.

While the traditional CDT scoring system revealed only poor sensitivity but excellent specificity in discriminating aMCI patients from healthy individuals, we found excellent sensitivity and a good specificity in discriminating these groups for in-air movements (i.e., time-in-air). Most interestingly, even in aMCI patients with normal cCDT score, usage of in-air trajectories yielded excellent sensitivity and a very good specificity in discriminating them from healthy individuals. This proportion of aMCI patients with normal cCDT was 80.0% of all aMCI patients. Thus, these findings indicate that even if clock drawing falls into the range of “normal” performance, this does not necessarily implicate that the subject is cognitively normal.

In line with the literature (Pinto and Peters, 2009), we obtained excellent sensitivity and specificity in distinguishing patients affected by eDAT from healthy individuals using the traditional CDT scoring system (Seigerschmidt et al., 2002; Pinto and Peters, 2009; Ehreke et al., 2011). However, time-in-air while performing clock drawing also demonstrated an excellent sensitivity and specificity to discriminate patients with eDAT from HCs.

Time-on-surface failed to differ between patients who are at risk of developing AD dementia (i.e., patients with aMCI) or are in the earliest stage of AD dementia (i.e., patients with eDAT) from healthy individuals. Furthermore, time to complete the whole CDT task (i.e., total-time) did not differ between patients with aMCI and eDAT. Thus, the variable best for use in screening for cognitive impairment and dementia is reflected in (non-visible) prolonged in-air trajectories.

The combined analysis of dCDT and cCDT variables did not improve the discrimination of patients with aMCI from controls in terms of sensitivity and specificity. Likewise, combining dCDT variables did not enhance diagnostic value in discriminating aMCI patients with unimpaired clock drawing performance from healthy individuals due to a lack of high diagnostic accuracy of total-time and time-on-surface.

A complex visuomotor task (e.g., clock drawing) requires the integration of cognitive information into a movement in the form of rules for guiding action (Smith et al., 2001). Thus, to perform clock drawing successfully it demands—apart of visual memory and reconstruction, and visuospatial abilities—executive functions (Shulman et al., 1986; Croisile, 1999; Forbes et al., 2004; Yan et al., 2008; Forbes-Mckay et al., 2014). Prolonged in-air trajectories might reflect impaired decision making due to frontal and temporoparietal disturbances that are often exhibited in the early course of AD and even in patients with aMCI (Samton et al., 2005; Zheng et al., 2012; Blanco Martín et al., 2016) and these interfere with cCDT demands like on-demand motor planning, execution (praxis) and executive function (Forbes et al., 2004; Yan et al., 2008; Forbes-Mckay et al., 2014). According to this we found significant impairment in TMT-B performance in our eDAT individuals compared to the other groups that might be most likely ascribed to increased deterioration in cognitive flexibility and executive functions (Crowe, 1998; Chen et al., 2009).

It is possibly due to the investigated subgroup of patients with an amnestic syndrome that there were—as expected—no significant differences in TMT-A and B performance between aMCI patients and HCs. However, between HCs and aMCI there were slight but non significant increased TMT-A and B scores observable. Thus, our data suggest that in contrast to time-in-air assessed with the dCDT, TMT-B might not be sensitive enough to assess cognitive processes captured by dCDT as early as on a subclinical level in aMCI populations (Buckner, 2004; Zheng et al., 2012). Indeed, dCDT demands seem to be much more complex than TMT-B requirements as dCDT impairments seem to be mainly determined by a reduced access to semantic memory about the appearance and functionality of a clock (i.e., semantic memory; Leyhe et al., 2009). However, the early detection of executive malfunction is of great interest as in line with our findings recent results suggest that patients with aMCI might additionally exhibit dysexecutive syndrome even in early stages that interfere with autonomy demands of these patients (Zheng et al., 2012; Blanco Martín et al., 2016).

The measurable discrepancies in the quality of movement execution between aMCI and HCs as well as in patients with eDAT and HCs have important clinical implications. Using a fine motor task such as drawing, the presented investigation supports the idea of using additional movement measures like time-in-air as an alternative diagnostic tool to enhance accuracy especially in those subjects with normal cCDT performance, indicating no cognitive impairment after visual inspection. Thus, the digitized assessment of one’s non-visible time-in-air movements can be used as supplementary information in identifying individuals in the predementia stage of AD.

A limitation of the presented study is the relatively small sample sizes of cognitively impaired subjects that may restrict validity and reliability of our conclusions. Additionally, the CDT (conventional or digital) assesses a variety of cognitive functions and thus does not allow conclusion which domain is affected. Further assessments are required to allow a valid interpretation of the results obtained by the CDT. Additionally, for or a more comprehensive use of this novel technique for screening purposes the implementation of additional features (e.g., assessment of velocity, pressure, writing thickness) might be potential and should be investigated in a broader clinical setting.

Examination of fine motor control such as drawing, contributes to the understanding of the full range of impairments displayed by patients with prominent memory dysfunction. The drawing task used in this study is easy to applicate by clinicians, the apparatus of digitizer is cost-effective and reliable, the computer software is user-friendly and customizable allowing its application as screening test for cognitive impairment in the context of AD in a broader clinical setting.

In conclusion, assessment of time-in-air using dCDT yielded a higher diagnostic accuracy for discrimination of aMCI patients from HC (78.0%) than the use of the traditional clock drawing (i.e., cCDT) scoring system (70.0%). Even in those a MCI patients with normal cCDT scores, assessment of time-in-air using dCDT yielded a clinically relevant diagnostic accuracy of 79.5%. Modern digitizing devices offer the opportunity to measure a broad range of visuo-constructive abilities that may be used as a fast and easy to perform screening instrument for the early and accurate detection of cognitive impairment in the context of AD.

## Author Contributions

SM, UE, PH and CL participated in study concept and design. SM, OP and CL participated in the acquisition or interpretation of data. SM and CL drafted the manuscript. OP and UE participated in the critical revision of the manuscript. SM did the statistical analysis.

## Conflict of Interest Statement

The authors declare that the research was conducted in the absence of any commercial or financial relationships that could be construed as a potential conflict of interest.
